# Targeted Therapy in Erdheim-Chester Disease: A Case Report and Review of the Literature

**DOI:** 10.7759/cureus.109423

**Published:** 2026-05-22

**Authors:** Ammar Alromdhan, Yagnapriya Ammakola, Gonzalo Cantu Soriano, Wasey Rehman, Joseph Fullmer, Mohammad Chisti

**Affiliations:** 1 Internal Medicine, Oakland University William Beaumont School of Medicine, Royal Oak, USA; 2 Internal Medicine, Corewell Health William Beaumont University Hospital, Royal Oak, USA; 3 Pathology and Laboratory Medicine, Corewell Health William Beaumont University Hospital, Royal Oak, USA; 4 Medicine, Oakland University William Beaumont School of Medicine, Rochester, USA; 5 Oncology, Corewell Health Southeast-Troy Campus, Troy, USA

**Keywords:** braf v600e, erdheim-chester disease, non-langerhans cell histiocytosis, targeted therapy, vemurafenib

## Abstract

Erdheim-Chester disease is a rare, aggressive non-Langerhans cell histiocytosis characterized by multisystem infiltration of histiocytes. It commonly affects middle-aged men and is driven by acquired mutations in the MAPK signaling pathway. This essential cellular pathway relays extracellular signals to the nucleus and regulates cell proliferation and survival.

We report the case of a 39-year-old male who presented with progressive, severe left-sided frontal and temporal headaches. Imaging revealed a symptomatic craniocervical mass, a nonsecreting pituitary lesion, and bilateral perinephric soft tissue infiltration extending throughout the retroperitoneum. Histopathologic evaluation demonstrated foamy histiocytes consistent with Erdheim-Chester disease, and molecular testing identified a *BRAF* mutation. Notably, no FDG-avid skeletal involvement was identified on available whole-body FDG PET/CT imaging, an uncommon feature in Erdheim-Chester disease. The patient was treated with the BRAF inhibitor vemurafenib, with dose reduction due to dermatologic toxicity, and subsequently tolerated therapy well. He has maintained a stable disease course at one-year follow-up. This report demonstrates the variable clinical manifestations of Erdheim-Chester disease and emphasizes the importance of molecular profiling in directing targeted therapeutic strategies.

## Introduction

Erdheim-Chester disease is a rare, aggressive non-Langerhans cell histiocytic neoplasm characterized by systemic infiltration of CD68-positive, CD1a-negative histiocytes [1]. It most commonly affects middle-aged adults, with a male predominance. This disease lacks a clearly established environmental, infectious, or hereditary etiology and is instead driven by acquired mutations in the MAPK pathway, an essential and widespread cellular pathway transmitting extracellular signals to the cell nucleus [1]. Relevant mutations include *BRAF* V600E, an amino acid substitution that constitutively activates the BRAF protein, which is identified in roughly 50-70% of cases and results in constitutive activation of the MAPK signaling cascade promoting histiocytic proliferation and survival, as well as *MAP2K1* mutations, which lead to constitutive, RAF-independent activation of the MEK1 protein kinase and downstream MAPK signaling [1,2]. A downstream effector within the RAS-RAF-MEK-ERK cascade, MEK1 is a protein kinase whose activation leads to the phosphorylation of ERK1/2 and persistent stimulation of histiocyte proliferation [3]. The shared consequence of both upstream mutation and constitutive MEK-ERK activation serves as the phenotypic basis for broad systemic involvement [3]. Multisystem involvement is characteristic of Erdheim-Chester disease, with manifestations including bilateral symmetric long bone osteosclerosis, retroperitoneal fibrosis with perirenal fat infiltration that may lead to hydronephrosis, as well as cardiovascular, retro-orbital, and central nervous system involvement [4]. Given the increasing presentation of Erdheim-Chester disease, with more than 1,500 cases reported as of 2019, its diagnosis and management remain clinically significant [4]. Traditionally, interferon alpha therapy was used to treat Erdheim-Chester disease; however, targeted therapies, such as BRAF and MEK inhibitors, have revolutionized the therapeutic landscape and significantly improved patient outcomes [5-7].

We report a case of a 39-year-old male with Erdheim-Chester disease who achieved a stable disease course with vemurafenib therapy. This report describes an atypical case of Erdheim-Chester disease without documented skeletal involvement and emphasizes the role of molecular profiling in guiding targeted therapy with BRAF inhibition.

## Case presentation

A 39-year-old male with a past medical history significant for type two diabetes mellitus, hypertension, and hypogonadism on testosterone replacement therapy presented with throbbing, moderate-to-severe headaches localized to the left frontal and temporal regions. The headaches were associated with lightheadedness, dizziness, and balance issues, but the patient denied diplopia, dysarthria, facial numbness, extremity numbness, or weakness. Neurologic examination was nonfocal, with intact mental status and cranial nerves, normal visual fields by finger confrontation, full strength throughout, intact sensation to light touch and pinprick, normal finger-to-nose testing, symmetric 2+ reflexes with downgoing toes, and intact gait without ataxia or Romberg sign. Despite treatment with Fioricet and acetazolamide (Diamox), his headaches progressively worsened. Initial laboratory evaluation was unremarkable. Given the progressive, refractory nature of his headaches, magnetic resonance imaging (MRI) of the brain with and without contrast was performed. Imaging revealed an enhancing lesion centered at the left aspect of the foramen magnum measuring approximately 2.8 × 2.7 × 2.8 cm, with associated mass effect on the cervicomedullary junction and rightward displacement of the midline, as shown in Figures 1A, 1B. The lesion demonstrated encasement of the intradural and intracranial segments of the left vertebral artery with preserved flow void. Additional enhancing lesions were identified involving the sella with suprasellar extension, measuring approximately 1.2 × 1.7 × 1.4 cm, as well as an enhancing extraconal lesion within the superior medial aspect of the left orbit measuring approximately 1.1 × 0.6 × 0.9 cm. Available endocrine evaluation documented normal thyroid-stimulating hormone (TSH), adrenocorticotropic hormone (ACTH), and cortisol levels, without documented evidence of thyroid or adrenal axis dysfunction.

**Figure 1:**
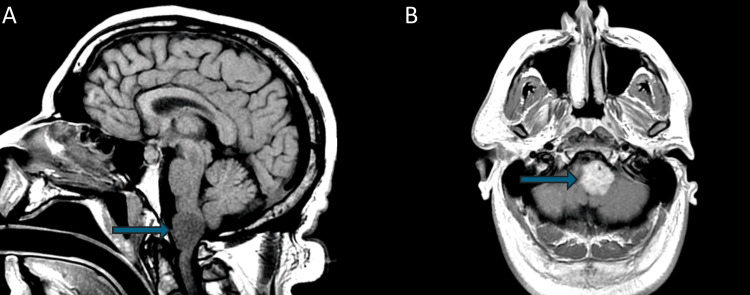
MRI of the foramen magnum lesion (A) Saggital T1-weighted MRI without contrast demonstrating a foramen magnum mass causing compression of the cervicomedullary junction (blue arrow). (B) Axial T1-weighted post-contrast MRI demonstrating a heterogeneously enhancing lesion centered at the left foramen magnum with rightward midline displacement (blue arrow).

Due to symptomatic cervicomedullary compression, the patient underwent a suboccipital craniotomy with C1 laminectomy for resection of the posterior fossa lesion. Histopathologic examination demonstrated a fibrohistiocytic process characterized by numerous foamy histiocytes within a fibrotic stroma on hematoxylin and eosin staining (Figure 2A). Immunohistochemistry showed a strong, diffuse CD163 positivity (Figure 2B), confirming a histiocytic lineage, with negative staining for CD1a and Langerin staining (Figures 2C, 2D), helping exclude Langerhans cell histiocytosis. S100 staining was also negative, arguing against Rosai-Dorfman disease. Together, these findings support a diagnosis of non-Langerhans cell histiocytosis, consistent with Erdheim-Chester disease.

**Figure 2:**
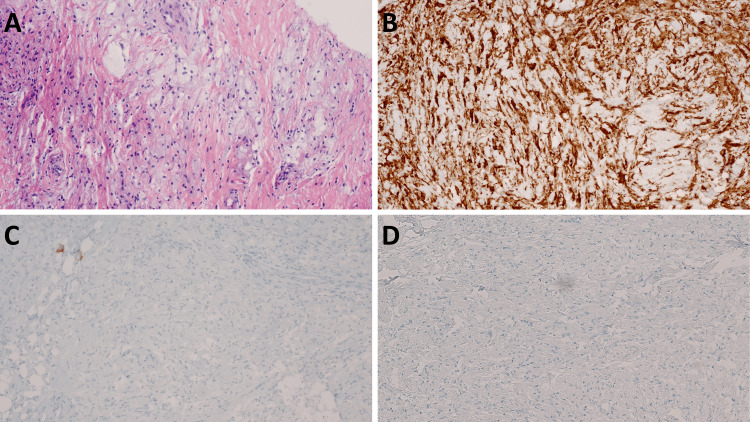
Histological and immunohistochemical findings (A) Hematoxylin and Eosin stain demonstrating a fibrohistiocytic infiltrate composed of numerous foamy histiocytes within a densely fibrotic stroma. (B) CD163 immunohistochemistry showing strong diffuse cytoplasmic positivity in histiocytes. (C) CD1a immunohistochemistry is negative. (D) Langerin immunohistochemistry is negative. All images are shown at 20x magnification.

In December 2023, computed tomography (CT) imaging of the abdomen and pelvis demonstrated extensive bilateral perinephric soft tissue encasing the renal vasculature with associated hydronephrosis and splenomegaly, as shown in Figures 3A, 3B. Renal function was preserved, with blood urea nitrogen 9 mg/dL, serum creatinine 0.60 mg/dL, and estimated glomerular filtration rate 126 mL/min/1.73 m². CT-guided core biopsy of the left perinephric mass was subsequently performed and revealed fibrous tissue with focal chronic inflammation and foamy histiocytic infiltration, without evidence of granuloma, abscess, or neoplasia (Figure 4). Immunohistochemistry was negative for epithelial markers (AE1/AE3) and renal lineage markers (PAX8). In the context of the confirmed histiocytic process in the posterior fossa lesion, these findings support systemic involvement by Erdheim-Chester disease.

**Figure 3:**
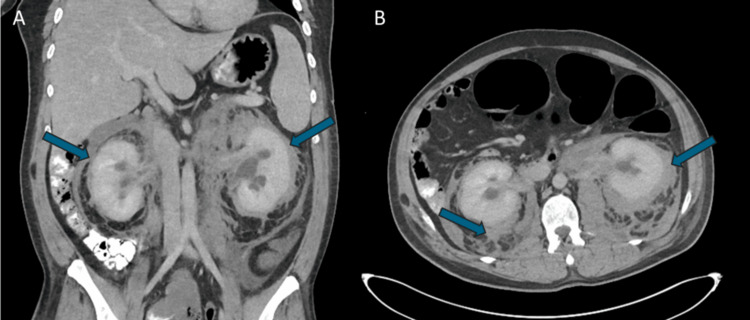
Computed tomography (CT) of the abdomen and pelvis (A) Coronal view demonstrating circumferential perirenal soft tissue encasement. (B) Axial view demonstrating similar findings, more pronounced on the left, producing the characteristic "hairy kidney" appearance. Blue arrows indicate bilateral perinephric soft tissue infiltration surrounding the kidneys.

**Figure 4:**
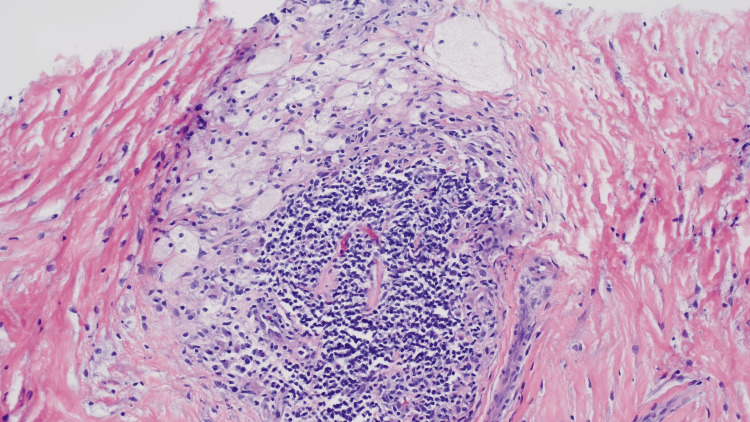
Histopathologic findings of perinephric infiltrate Hematoxylin and Eosin staining of the left perinephric mass showing scattered cells morphologically consistent with foamy histiocytes within fibrotic tissue, at 20× magnification.

Fluorodeoxyglucose (FDG) positron emission tomography/computed tomography (PET/CT) demonstrated mild FDG uptake within the extensive bilateral perinephric soft tissue, with a maximum standardized uptake value (SUVmax) of up to 2.4, as well as metabolically active lesions involving the cervicomedullary junction and pituitary region, with SUVmax values of 12.3 and 12.7, respectively. Subsequent next-generation sequencing was performed via liquid biopsy per the treating team's clinical preference and identified a *BRAF* V600E mutation. The patient was initiated on vemurafenib 960 mg twice daily (BID). After approximately 10 days of therapy, he developed a diffuse maculopapular rash, prompting temporary discontinuation of vemurafenib and treatment with prednisone 50 mg daily for five days. Vemurafenib was subsequently restarted at a 50% dose reduction of 480 mg BID. He experienced intermittent rash flares thereafter, which were successfully managed with short courses of prednisone and hydroxyzine. Follow-up CT imaging of the abdomen revealed stable disease following the initiation of vemurafenib, with extensive retroperitoneal soft tissue infiltrative changes involving both the perinephric and perivascular spaces and mild symmetric bilateral hydronephrosis. Follow-up brain MRI performed more than one year after therapy demonstrated an interval decrease in the residual extra-axial mass at the foramen magnum, with mildly improved mass effect on the left medulla and less avid enhancement. The MRI also showed decreased prominence of the pituitary infundibulum and pituitary gland, with no new brain parenchymal signal abnormality, diffusion restriction, or pathologic enhancement. Follow-up PET/CT was not available; therefore, metabolic response could not be assessed.

## Discussion

Erdheim-Chester disease is heterogeneous in presentation, with skeletal involvement reported in nearly all patients, most commonly manifesting as bilateral symmetric osteosclerosis of the long bones [8]. Extraosseous findings include cardiac, pulmonary, renal, and pituitary manifestations, occurring in approximately half of patients [9]. In contrast, our patient presented with prominent neurological and renal symptoms in the absence of significant skeletal involvement, an atypical presentation that can delay recognition of the disease.

An important component in guiding therapy is molecular profiling, including genetic sequencing to determine whether the patient harbors mutations activating the MAPK pathway, which are found in more than 80% of patients with Erdheim-Chester disease, with *BRAF* V600E identified in approximately 57-70% of cases [4]. *BRAF* V600E encodes a constitutively active kinase that promotes persistent activation of the MAPK signaling cascade (RAS-RAF-MEK-ERK), resulting in nuclear translocation of ERK and transcription of genes promoting cell proliferation and survival [10]. In these patients, BRAF inhibitors, such as vemurafenib and dabrafenib, have been shown to improve survival outcomes [11].

For the rare subset of patients (<1%) with Erdheim-Chester disease harboring activating mutations in CSF1R, a receptor tyrosine kinase that serves as an alternative oncogenic driver distinct from MAPK pathway mutations, treatment with the CSF1R inhibitor pexidartinib remains an effective option [12]. In our patient, vemurafenib therapy was initiated following identification of a *BRAF* V600E mutation.

In the phase II VE-BASKET trial, which led to FDA approval of vemurafenib for Erdheim-Chester disease, vemurafenib was administered at a dose of 960 mg twice daily and demonstrated significant clinical efficacy, with an overall response rate of 54.5%, a two-year progression-free survival of 86% (95% CI, 72-100%), and a two-year overall survival of 96% (95% CI, 87-100%). Notably, all treated patients achieved a complete metabolic response on PET/CT imaging. The most commonly reported adverse effects included arthralgias, maculopapular rash, alopecia, and hyperkeratosis [11].

Despite favorable outcomes, dose reductions are frequently required in Erdheim-Chester disease to balance disease control with treatment-related toxicity. One study reported a high response rate of 85% with BRAF inhibitor therapy but observed treatment discontinuation in 61% of patients at a median follow-up of four years, primarily due to adverse events and diminished quality of life [13]. Similarly, Cohen et al. demonstrated that 75% of patients relapsed within 6 months of treatment discontinuation, with clinical improvement observed upon resumption of therapy [14]. Reiner et al. further reported disease relapse in 77% of patients following interruption of BRAF or MEK inhibitors, even among those who had achieved complete or partial metabolic responses on FDG-PET imaging [15]. These findings support dose reduction rather than discontinuation to maintain disease control while minimizing toxicity, as demonstrated in our patient.

A retrospective study conducted at Memorial Sloan Kettering Cancer Center demonstrated that dabrafenib can be used as an alternative in patients who do not tolerate vemurafenib. In this study, 11 patients treated with dabrafenib achieved response rates comparable to vemurafenib but experienced reduced toxicity, particularly fewer cutaneous adverse effects [16]. This provides an important alternative for patients experiencing intolerable adverse effects, such as the maculopapular rash observed in our patient.

Cobimetinib is a MEK1/2 inhibitor that is highly effective in Erdheim-Chester disease regardless of genotype, as MEK1/2 represents the obligate downstream target of all MAPK pathway mutations [17]. Trametinib is an alternative MEK1/2 inhibitor that can be used interchangeably with cobimetinib in patients with MAPK pathway mutations [18]. Combination therapy with BRAF and MEK inhibitors (such as dabrafenib plus trametinib) has been used in select cases. A notable case reported by Nordmann et al. described a patient with *BRAF* V600E-positive Erdheim-Chester disease who developed acquired resistance to dabrafenib through the emergence of a *KRAS* mutation in a new lesion. Addition of a MEK inhibitor (trametinib) successfully overcame resistance, demonstrating that combination BRAF/MEK inhibition may be an effective strategy in cases of acquired resistance to BRAF inhibitor monotherapy [19]. Together, these findings underscore the role of next-generation sequencing in identifying molecular drivers that inform personalized treatment strategies.

Neurological involvement is a well-recognized manifestation of Erdheim-Chester disease and is frequently associated with more aggressive disease courses. In patients with central nervous system involvement, traditional therapies, such as interferon-alpha, may demonstrate limited efficacy, further reinforcing the importance of molecular profiling through next-generation sequencing to guide the selection of targeted therapeutic strategies.

## Conclusions

This case demonstrates an atypical presentation with prominent neurological and renal manifestations in the absence of classic skeletal involvement, emphasizing the importance of maintaining diagnostic consideration for Erdheim-Chester disease even in nonclassic clinical scenarios. Given the central role of MAPK pathway dysregulation in disease pathogenesis, next-generation sequencing is crucial for identifying actionable molecular drivers and guiding targeted treatment selection. As demonstrated by the clinical course of our patient, targeted therapies, such as BRAF and MEK inhibitors, can achieve durable disease control, even when dose adjustments are required to manage treatment-related toxicity.
